# Hyperechogenicity of the Substantia Nigra in Parkinson's Disease: Insights from Two Brothers with Markedly Different Disease Durations

**DOI:** 10.1155/2017/3673159

**Published:** 2017-01-11

**Authors:** Julie M. Hall, Matthew J. Georgiades, Deborah A. Hammond, Xiaoting Feng, Ahmed A. Moustafa, Simon J. G. Lewis, Gabrielle Todd

**Affiliations:** ^1^Parkinson's Disease Research Clinic, Brain and Mind Centre, University of Sydney, 100 Church St, Camperdown, NSW, Australia; ^2^School of Social Sciences and Psychology, Western Sydney University, Horsley Road & Bullecourt Road, Milperra, NSW 2214, Australia; ^3^Marcs Institute for Brain and Behaviour, Western Sydney University, Horsley Road & Bullecourt Road, Milperra, NSW 2214, Australia; ^4^School of Pharmacy and Medical Sciences and Sansom Institute for Health Research, University of South Australia, P.O. Box 2471, Adelaide, SA 5001, Australia

## Abstract

We present clinical features and substantia nigra morphology for two brothers with Parkinson's disease (PD) aged 60 and 59 years. The brothers were diagnosed at 41 and 50 years of age, respectively. Both patients exhibited an abnormally large area of substantia nigra echogenicity bilaterally when viewed with transcranial ultrasound. The abnormality was similar in both brothers despite one having a much longer disease duration than the other. These findings further highlight that transcranial ultrasound is not associated with severity of clinical symptoms, but it might assist in the diagnosis of PD provided that it is combined with other variables known to precede PD.

## 1. Background

Transcranial sonography (TCS) is a noninvasive diagnostic imaging technique that allows scanning of brain parenchyma in two-dimensional black and white slices. The morphology of the substantia nigra (SN) can be assessed with TCS in humans. In 90% of individuals with Parkinson's disease (PD) the area of echogenicity at the anatomical site of the SN is abnormally large (e.g., >0.20 cm^2^; SN+) [1] compared to just 10% of age-matched controls and patients with atypical Parkinsonian disorders [2]. There also appears to be a genetic susceptibility for SN+ in relatives of PD patients [3]. Here, we report for the first time TCS findings in two brothers with diagnosed PD.

## 2. Case Report

Both patients underwent disease characterization using the following assessments: revised Movement Disorder Society-Unified Parkinson's Disease Rating Scale (MDS-UPDRS) including the clinical Hoehn & Yahr (H&Y) motor scale, the Mini Mental State Examination (MMSE), revised Beck's Depression Inventory (BDI-II), and REM Sleep Behaviour Disorder Questionnaire (RBDQ). Age, disease duration, and Levodopa equivalent dose were obtained. TCS was performed with an Esaote MyLab Seven ultrasound system (penetration depth: 16 cm; dynamic range: 49 dB) equipped with a 2.5 MHz phased array transducer (SP 2430, Esaote, Genova, Italy). The examination was performed bilaterally through the preauricular acoustic bone window by one experienced operator (GT) following established protocols [1]. Written consent was obtained from both patients and the local ethics committee approved the study.

### 2.1. Case 1

Patient 1 was a 60-year-old, right-handed male with a 19-year history of PD. He presented with left-sided symptoms and was Hoehn and Yahr Stage II with mild motor fluctuations. Patient 1 had a MDS-UPDRS total score of 44 and motor subscore of 34 (see Table 1). No significant cognitive impairment, affective symptoms, freezing of gait, or REM sleep behaviour disorder was observed in Patient 1.

#### 2.1.1. Transcranial Sonography

**Figure 1:**
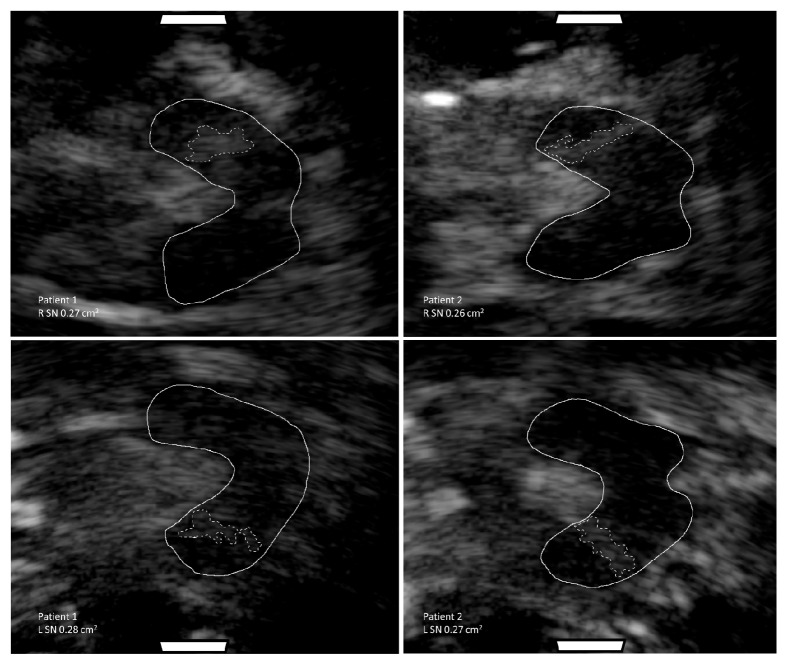
Transverse view of substantia nigra (SN) morphology in Patients 1 and 2 using transcranial sonography. Images of the right (top) and left (bottom) SN are shown. Solid line encircles the mesencephalic brainstem. Dotted line encircles the SN. White rectangles correspond to the placement of the probe.

The bone windows of Patient 1 were subjectively rated as excellent. The area of SN echogenicity was abnormally large bilaterally [4] (Figure 1), with measurements of 0.27 cm^2^ (right) and 0.28 cm^2^ (left). The diameter of the third ventricle was slightly enlarged, measured at 0.65 cm (Table 1). The raphe nucleus was uninterrupted and rated as normal.

### 2.2. Case 2

Patient 2, the younger brother of Patient 1, was a 59-year-old, left-handed male diagnosed with PD 9 years ago. Patient 2 also presented with left-sided symptoms and had Hoehn and Yahr Stage II with mild motor fluctuations. Patient 2 had a MDS-UPDRS total score of 19 and motor subscore of 12 (Table 1). Similar to Patient 1, Patient 2 had no significant cognitive impairment, affective symptoms, freezing of gait, or REM sleep behaviour disorder.

#### 2.2.1. Transcranial Sonography

Both the left and right bone window of Patient 2 was subjectively rated as excellent. Sonographic measurements of the area of SN echogenicity were abnormally large [4]: 0.26 cm^2^ on the right and 0.27 cm^2^ on the left side. The diameter of the third ventricle was 0.31 cm and was rated as normal (Table 1). The raphe nucleus appeared uninterrupted and was rated as normal.

## 3. Discussion

This is the first study to report TCS findings in two similarly aged brothers with PD.

Neither patient had undergone genetic testing but there was no other family history of PD or Parkinsonism. Both patients had a comparable phenotype with left-sided onset, mild motor fluctuations, absence of freezing of gait, preserved cognition, and no affective symptoms or REM sleep behaviour disorder. However, they differed markedly in their durations of disease and severity of motor signs and symptoms. Nevertheless, the area of SN echogenicity was strikingly similar although the shape of the SN differed between patients. This variance in shape may arise from differences in iron deposition, neuromelanin, and microglia activation; mechanisms that are believed to contribute to increased area of SN echogenicity [5, 6]. Despite the known topographical midbrain pathology and arrangement of the projections of the SN pars compacta to the striatum [7], little is known about the clinical association with the shape of the enlarged SN signal. The current data supports previous studies that report lack of a correlation between SN echomorphology and disease duration and symptom severity [8]. It has been suggested that SN+ is a marker for nigral cell injury and factors leading to SN+ might be of significance in the pathogenesis of PD [1]. Early detection of an increased SN echogenicity area could allow early intervention to prevent nigrostriatal cell loss when therapeutic strategies are available.

Interestingly, a five-year follow-up study showed a stronger correlation between the SN morphology and motor severity, suggesting that the area of SN echogenicity in the earlier stages of disease could be used as a prognostic tool for more marked disease severity in the advanced stages [9]. If this were true, then the findings of the current study could especially be of importance for Patient 2, the younger brother with shorter disease duration.

Healthy older adults with the abnormality are 17 times more likely to develop PD over a 3-year period [10]. However, whilst 45% of first-degree relatives exhibit the increased echogenicity signal [3], only a small percentage of SN+ individuals will ultimately develop PD. Thus TCS provides a unique opportunity for future investigation of PD risk factors, protective influences for developing PD, and possibly early disease mechanisms in at risk populations, provided that it will be combined with other known biomarkers of PD, such as smell, colour vision, and REM sleep behaviour disorder.

It should be noted that categorisation of SN echomorphology is specific to the ultrasound model used and population tested and is based on normative data for a large sample of healthy individuals. However, such data is not yet available for the ultrasound system used in the current study. Preliminary normative data is available for other Esaote models with marked hyperechogenicity defined as >0.25 cm^2^ [11]. The area of SN echogenicity measured in the current study ranged from 0.26–0.28 cm^2^. Thus, the two brothers likely exhibited marked hyperechogenicity.

## Figures and Tables

**Table 1:** Demographical and clinical characteristics.

	Patient 1	Patient 2
Age (years)	60	59
Time since diagnosis (years)	19	9
LED (mg/day)	500	625
H&Y	2	2
MDS-UPRDS total	44	19
MDS-UPDRS part III	34	12
MMSE	30	30
BDI-II	1	6
RBDQ	5	3
Area of SN echogenicity (cm^2^)	*R* = 0.27, *L* = 0.28	*R* = 0.26, *L* = 0.27
Diameter of third ventricle (cm)	0.65	0.31

H&Y: Hoehn and Yahr Clinical staging; MDS-UPDRS: Movement Disorder Society-Unified Parkinson's Disease Rating Scale; LED: Levodopa equivalent dose; MMSE: Mini Mental State Examination; BDI-II: Beck Depression Inventory-II; RBDQ: REM Sleep Behaviour Disorder Questionnaire.
